# Hypersensitivity reactions to biologic disease modifying antirheumatic drugs: experience from the TTSH Singapore biologics registry

**DOI:** 10.1186/2045-7022-4-S3-P54

**Published:** 2014-07-18

**Authors:** Bernard Thong, Wei-Joo Choy, Ee-Ling Ng, Xanthe Chua, Kok-Ooi Kong, Weng-Giap Law, Hwee-Siew Howe

**Affiliations:** 1 Tan Tock Seng Hospital, Rheumatology, Allergy and Immunology, Singapore

## Background

To describe the prevalence and risk factors for hypersensitivity reactions (HSR) to biologic disease modifying antirheumatic drugs (bDMARD) from the TTSH Singapore Biologics Registry.

## Methods

Consecutive patients who had ever received a bDMARD were followed up during the study period 1 January 2001 to 31 December 2013.

## Results

There were 217 patients of Asian ethnicity who had ever received a bDMARD, of whom 30 (13.8%) had received more than 1 bDMARD. These comprised rituximab (78, 35.9%), infliximab (59, 27.2%), etanercept (51, 23.5%), adalimumab (41, 18.9%), golimumab (8, 3.7%), anakinra (4, 1.8%), abatacept and tocilizumab (3, 1.4% each). HSR occurred in 4 female patients following previous exposure to an anti-tumour necrosis factor inhibitor (TNFi) (2) and rituximab (2) respectively. A 67-year-old Chinese female with rheumatoid arthritis developed fever and hypotension without rash or other organ-specific involvement, within 1 hour of the second dose of infliximab 200 mg 2 weeks later. She switched to adalimumab without adverse reaction. Another 34-year-old Malay female with psoriatic arthritis developed optic neuritis following the third dose of adalimumab 40 mg every other week. She recovered and switched to abatacept without adverse reaction. A 28-year-old female with systemic lupus erythematosus (SLE) developed acute urticaria following the second dose of the third course of rituximab 500 mg given 2 weeks apart. Another 27-year old female with SLE developed anaphylaxis with flushing, facial angioedema, dyspnoea and wheeze, following the second dose of the second course of rituximab 500 mg 5 years later. None of the patients with immediate reactions to infliximab/ rituximab agreed to have diagnostic skin testing in view of costs. The patient with anaphylaxis declined desensitization to rituximab. There were no HSR to any of the other bDMARDs. Exposure to more than 1 bDMARD within/across different classes did not increase the risk of HSR. There were no deaths from HSR.

## Conclusion

HSR to bDMARDs is uncommon in our cohort. Risk factors include repeated dosing, but not exposure to more than 1 bDMARD within/across different classes.

